# Rapid Glass‐Substrate Digital Light 3D Printing Enables Anatomically Accurate Stroke Patient‐Specific Carotid Artery‐on‐Chips for Personalized Thrombosis Investigation

**DOI:** 10.1002/adma.202508890

**Published:** 2025-09-11

**Authors:** Yunduo Charles Zhao, Zihao Wang, Arian Nasser, Allan Sun, Zhao Wang, Yingqi Zhang, Jianfang Ren, Haimei Zhao, Nicole Alexis Yap, Yinyan Wang, Zhiyong Li, Ken S Butcher, Freda Passam, Timothy Ang, Lining Arnold Ju

**Affiliations:** ^1^ School of Biomedical Engineering The University of Sydney Darlington NSW 2008 Australia; ^2^ Charles Perkins Centre The University of Sydney Camperdown NSW 2006 Australia; ^3^ The University of Sydney Nano Institute (Sydney Nano) The University of Sydney Camperdown NSW 2006 Australia; ^4^ Heart Research Institute Newtown NSW 2042 Australia; ^5^ Department of Neurosurgery Beijing Tiantan Hospital Capital Medical University Beijing 100070 China; ^6^ Brain Health Group The George Institute for Global Health Barangaroo NSW 2000 Australia; ^7^ School of Mechanical Medical and Process Engineering Faculty of Engineering Queensland University of Technology Brisbane 4001 Australia; ^8^ Prince of Wales Clinical School Prince of Wales Hospital Randwick NSW 2031 Australia; ^9^ Department of Haematology Royal Prince Alfred Hospital Camperdown NSW 2006 Australia; ^10^ Central Clinical School Faculty Medicine and Health The University of Sydney Camperdown NSW 2006 Australia; ^11^ Departments of Interventional Neuroradiology Neurology Royal Prince Alfred Hospital Camperdown NSW 2006 Australia; ^12^ Department of Engineering University of Cambridge Cambridge CB2 1TN UK

**Keywords:** 3D printing, biofabrication, organ‐on‐a‐chip, stroke, thrombosis

## Abstract

Translating patient‐specific vascular geometries into functional microfluidic devices remains challenging due to fabrication limitations and lengthy processing times. Here, an ultrafast microfabrication platform is introduced using glass‐substrate digital light processing 3D printing for creating patient‐specific carotid artery‐on‐a‐chip devices. The optimized protocol employs treated glass slides as printing substrates and custom‐designed mechanical clamping, reducing manufacturing time from over 10 h to under 2 h with ≈100% success rate. The system accurately reproduces complex anatomical features from CT angiography data of stroke patients, including stenoses, bifurcations, and ulcerations that conventional reconstruction methods often miss. Computational fluid dynamics validation confirms preserved hemodynamic similarity between patient‐scale and chip‐scale geometries, with matched wall shear rates maintaining physiological relevance despite 30‐fold size reduction. The platform supports endothelialization and blood perfusion, enabling real‐time visualization of thrombotic processes. Integration with laser ablation technology allows controlled endothelial injury modeling at patient‐specific vulnerable sites. Quantitative analysis reveals 7–10‐fold higher platelet translocation in the high shear zone (>1000 s^−1^), demonstrating the platform's capability to capture shear‐dependent thrombotic mechanisms. This rapid biomanufacturing approach represents a significant advance in patient‐specific organ‐on‐a‐chip technology, with applications in personalized medicine and vascular device development.

## Graphical abstract

A rapid glass‐substrate 3D microfabrication method creates anatomically accurate carotid artery‐on‐a‐chip models from stroke patient scans in 2 h. These miniaturized devices replicate patient‐specific blood vessel geometry and flow patterns, enabling real‐time imaging of clot formation under controlled injury. This rapid biomanufacturing approach represents a significant advance in patient‐specific organ‐on‐a‐chip technology, with applications in personalized medicine and vascular device development.

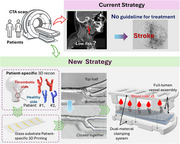

## 1 Introduction

**Figure 1:**
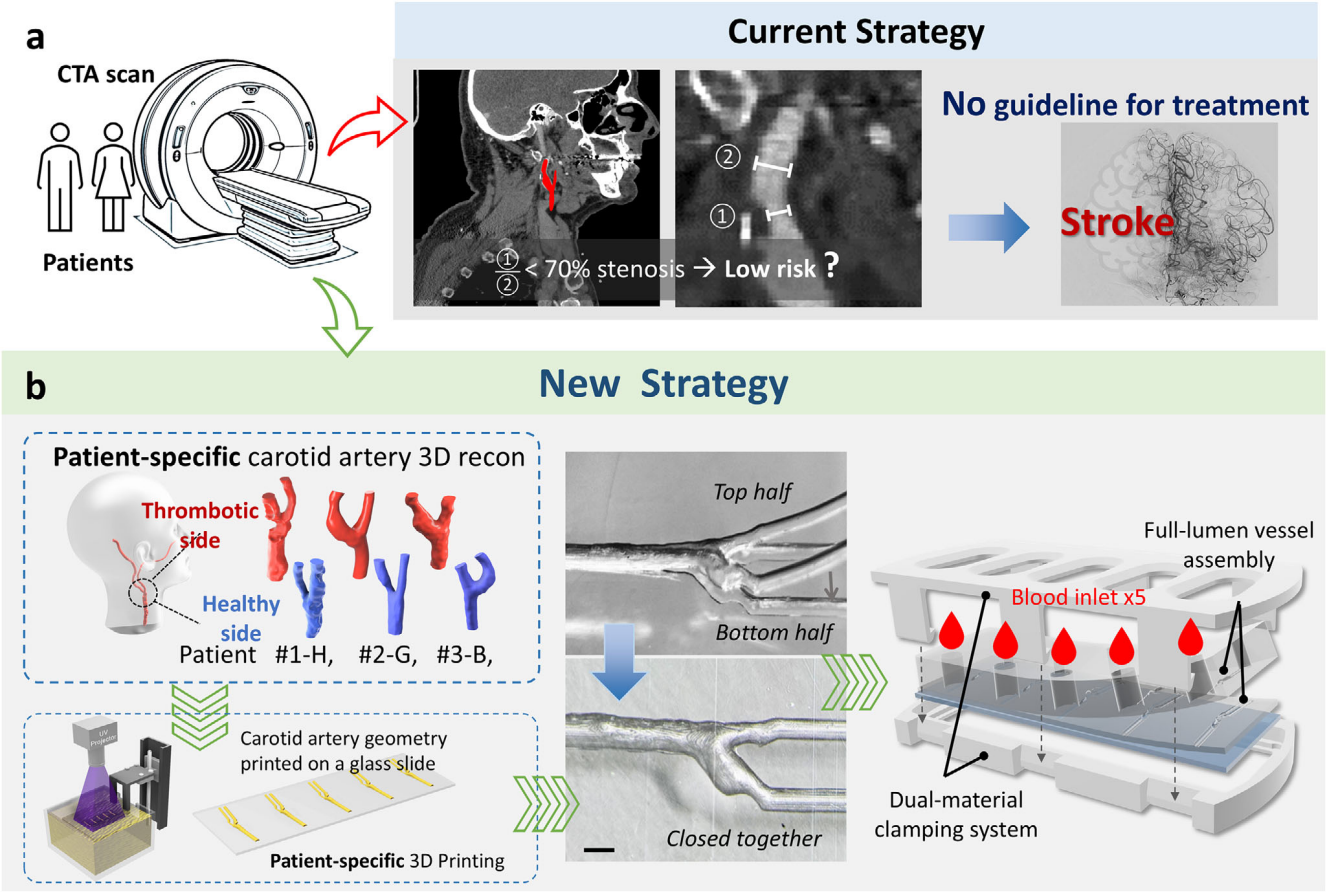
Microprecision 3D fabrication of patient‐specific Carotid Artery‐Chip for personalized thrombotic assessment. a) The current strategy to assess stroke risk relies on measuring carotid artery stenosis. *Left*: Patients undergo CTA imaging of carotid arteries to diagnose the cause of stroke and determine treatment strategies. *Middle*: CTA images are used to measure stenosis levels, with stenosis defined as the ratio of the narrowed vessel diameter (①) to the downstream normal vessel diameter (②). *Right*: A digital subtraction angiography (DSA) image from an example patient shows less than 70% stenosis with no guideline for treatment, yet the patient eventually developed a stroke. b) Clinical 3D reconstruction of carotid arteries for stroke patients. *Left*: CTA images are utilized to reconstruct patient‐specific carotid artery geometries, including both thrombotic and healthy sides, identified by a number and a letter. *Middle*: The alignment and assembly of the two halves of the patient‐specific Carotid Artery‐Chip. Scale bars = 200 µm. *Right*: The final chip design for personalized thrombotic risk assessment, using a clamp system to mechanically bond the two halves together. The top half is punched with one inlet (*d* = 6 mm) and one outlet (*d* = 1 mm) for each channel to allow blood perfusion.

The role of the carotid artery, particularly its internal branch, in the pathophysiology of acute stroke remains a complex and multifaceted challenge.^[^ ^1^, ^2^ ^]^ Both initial and recurring strokes often stem from sophisticated thrombotic events within the carotid artery, yet the exact progression from long‐term stenosis to sudden stroke continues to challenge our understanding.^[^ ^3^, ^4^ ^]^ Current diagnostic tools, such as Computed Tomography Angiography (CTA), excel at identifying vascular abnormalities after strokes occur but fall short in providing insights into the dynamic processes that lead to thrombosis.^[^ ^1^, ^5^ ^]^ These imaging methods provide valuable anatomical and blood flow snapshots but lack the resolution and functional specificity needed to accurately assess the interplay of elements described by Virchow's Triad: altered blood flow, endothelial damage, and increased blood coagulability. Current clinical guidelines, which primarily focus on measuring stenosis levels, have limitations in risk assessment (**Figure** **1**a). In many cases, patients with stenosis levels below 70% are classified as “low risk,” and no treatment is recommended.^[^ ^6^ ^]^ However, some of these “low risk” patients still go on to develop strokes (Figure 1a), suggesting that factors beyond simple stenosis measurements influence thrombotic risk.

Understanding the mechanistic basis of thrombosis in patient‐specific vascular contexts requires experimental platforms that can accurately replicate both the complex anatomical features and hemodynamic environments of individual patients.^[^ ^7^, ^8^ ^]^ In vitro microfluidic approaches have emerged as promising tools for studying vascular biology and thrombosis under controlled conditions.^[^ ^9^, ^10^, ^11^ ^]^ However, conventional microfluidic designs typically employ simplified channel geometries that fail to capture the intricate anatomical features of patient vasculature that significantly influence local hemodynamics and subsequent thrombotic processes.^[^ ^9^, ^12^ ^]^ Recent advances in digital light processing (DLP) based micro‐precision 3D printing have opened new possibilities for creating complex vessel‐on‐a‐chip devices.^[^ ^13^, ^14^ ^]^ This technology enables direct fabrication of 3D structures with high resolution and rapid processing time.^[^ ^15^ ^]^ However, translating patient‐specific vascular imaging data into functional microfluidic devices while maintaining anatomical accuracy at the microscale has remained challenging. Furthermore, ensuring proper surface chemistry and biocompatibility for endothelialization and blood perfusion requires careful consideration of materials and surface modification strategies.

Here, we present an innovative approach combining DLP‐based micro‐precision 3D printing with computational fluid dynamics to create anatomically accurate, real stroke patient‐specific Carotid Artery‐Chips. Our fabrication system enables rapid production of microfluidic devices that precisely replicate individual patients' carotid artery geometries, including stenotic regions and bifurcations. The platform integrates several key innovations: an ultrafast fabrication process that accurately translates patient‐specific vascular imaging data into functional devices, computational modeling that enables the scaling of physiological flow conditions to microfluidic dimensions while maintaining relevant hemodynamic parameters, and a novel laser injury model for studying localized thrombosis under controlled conditions.

The system's design incorporates insights from recent advances in thrombosis mechanobiology research and therapeutics,^[^ ^8^ ^]^ enabling investigation of how specific anatomical features and flow patterns influence platelet activation, adhesion, and thrombus formation. By combining precise geometric control with the ability to maintain stable endothelialization and support blood perfusion under physiologically and pathologically relevant flow conditions, our platform provides new opportunities for studying the mechanical aspects of thrombosis in anatomically accurate contexts, potentially offering insights into why some patients with apparently low‐risk stenosis levels still develop thrombotic complications.

## 2 Results

### 2.1 Rapid Prototyping and Optimization of Patient‐Specific Carotid Artery‐Chips

We developed an innovative, ultrafast, and highly accurate fabrication system for patient‐specific Carotid Artery‐Chips to address the limitations of traditional vessel modeling. Conventional arterial models often struggle to accurately replicate the high flow rates and delicate structures of carotid arteries while maintaining practical blood volume requirements. To overcome these challenges, we employed a DLP‐based 3D printer, BMF S240. This high‐performance printing enabled the construction of detailed carotid artery models suitable for microfluidic testing,^[^ ^16^ ^]^ significantly surpassing the capabilities of common 3D printers limited to minimum printing features of 100 µm (Figure 1).

Our process began with the extraction of CTA carotid artery images of three patients clinically diagnosed with stroke, totaling five carotid artery geometries from bilateral arteries of two patients, plus one unilateral from the third patient (Figure 1b, *left*). These images were meticulously scaled down to achieve a common carotid artery luminal diameter of 200–300 µm, making them compatible with microfluidic applications. To ensure anatomical accuracy, we employed a custom surface smoothing algorithm coupled with manual refinements.^[^ ^17^ ^]^ This approach was crucial for preserving essential anatomical features, particularly in diseased regions where precise replication of stenosis and ulcerations was vital for accurate modeling.

The reconstructed vessel geometries were then split into two halves along the middle axial plane to facilitate their conversion into functional microfluidic devices (Figure 1b, *left*). The preprocessing stage for 3D printing involved a meticulous procedure of cleaning and treating glass slides to serve as printing substrates (Figure S1, Supporting Information) to eliminate the need for a bulky base, thereby enhancing the surface finish of the fabrication (Figure S2, Supporting Information), increasing the overall success rate (Figure S3, Supporting Information), and dramatically reducing the print time from over 10 h to ≈2 h.

To ensure precise positioning of the glass slide on the build platform, we designed and 3D printed a custom locator composed of four L‐shaped brackets (Figure S4, Supporting Information). This innovation allowed for consistent and accurate placement of the substrate, which is critical for maintaining the high fidelity of the printed models (Video S1, Supplementary Video1). To produce the full lumen PDMS chip, we followed a previously described procedure^[^ ^18^ ^]^ with an aluminum frame to standardize the chip's shape and thickness (Figure S5, Supporting Information). The PDMS mixture was then cured (Video S2, Supplementary Video2) and fabricated (Video S3, Supplementary Video3) into microfluidic channels following the standard procedure.^[^ ^18^ ^]^

A key innovation in our fabrication process was the development of a mechanical clamp system designed to prevent potential leakage and separation during chip handling and operation (Figure 1b, *right*). After assembly and clamping, each chip underwent a sterilization process (Video S4, Supplementary Video4).

Our optimized fabrication method significantly reduced manufacturing time to less than 2 h per mold, with an impressive success rate of ≈100%. This high efficiency and reliability make the process scalable and highly suitable for producing high‐fidelity carotid artery models for thrombotic assessment (Figure 1b).

### 2.2 Carotid Artery‐Chip Fluid Flow Characterization at Microfluidic Scale

**Figure 2:**
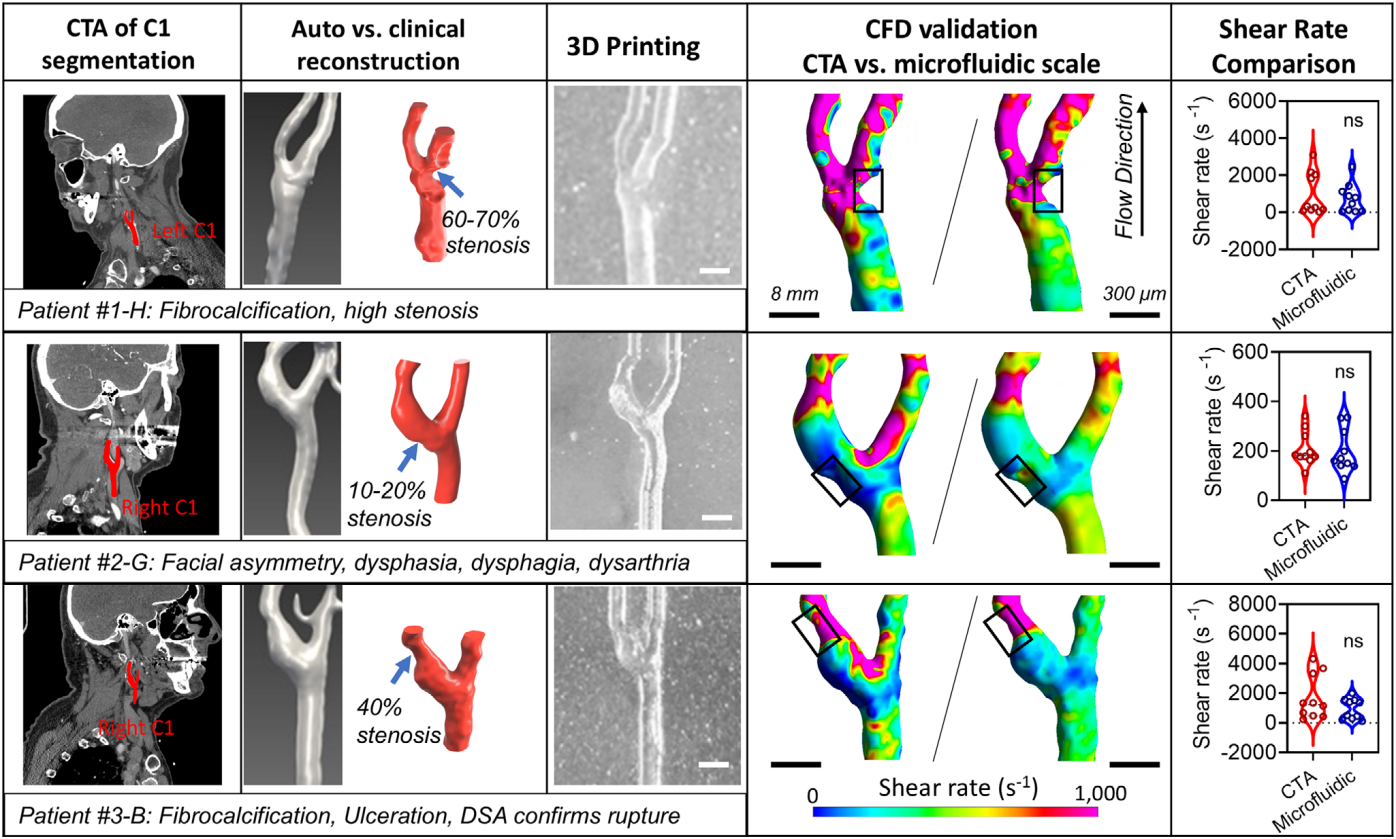
Detailed 3D reconstruction and fabrication of patient‐specific carotid artery vessel geometries. *1st column*: The carotid arteries of patients #1‐3, diagnosed as pathological and potential triggers for strokes, were subjected to CTA imaging to confirm their pathologies. *2nd column*: The clinical reconstruction, enhanced by incorporating clinical insights, significantly improved the detail and realism of the vessel geometries, ensuring precise fabrication. *3rd column*: The differential interference contrast (DIC) microscope image illustrates the detailed top half of the printed mold. This scaling was optimized by testing the DLP printer to balance blood consumption for subsequent experiments with fabrication precision. *4th column*: Computational fluid dynamics simulations validated that the scaled‐down designs preserved the shear rate across the vessel bifurcation area, ensuring the accuracy and relevance of the models for further experimental analysis. *5^th^ column*: Quantitative comparison of shear rates in the stenotic zone (black box region in the 4^th^ column) between CTA and microfluidic scales showed no statistically significant differences (ns), validating the geometric downscaling for experimental modeling.

The foundation of our Carotid Artery‐Chip model lies in the accurate replication of patient‐specific vessel geometries. CTA images, enhanced by an iodine‐based contrast agent administered through intravenous injection, provided detailed visualization of the arterial anatomy (**Figure** **2**, *1st column*). While most modern CT scanners offer automatic 3D reconstruction capabilities (Figure 2, *2nd column*), we found that these automated processes often failed to capture crucial details of the blood vessel lumen, particularly in cases with ulcerations or complex stenoses (Figure 2, *2nd column*).

To address this limitation, we developed a refined 3D reconstruction approach in collaboration with experienced neurologists. This method ensured precise delineation of the 3D structure of the carotid bifurcation, a critical region where the common carotid artery (CCA) divides into the internal carotid artery (ICA), supplying blood to the brain, and the external carotid artery (ECA), providing blood to the face. By manually reconstructing this crucial zone, we retained intricate anatomical details necessary for accurate modeling and subsequent data analysis (Figure 2, *2nd column*).

Using these precisely obtained 3D images of the carotid artery (Experimental Section), we fabricated molds that faithfully replicated the patient‐specific vessel geometries (Figure 2, *3rd column*). To ensure that the hemodynamics at the microfluidic scale were comparable to the in vivo CTA scale of the patients, we conducted comprehensive computational fluid dynamics (CFD) analyses following established methods^[^ ^18^, ^19^, ^20^ ^]^ (Figure 2, *4th column*).

These CFD analyses were instrumental in mapping shear rate distributions, a key hemodynamic factor in thrombosis development, at both the microfluidic and physiological CTA scales, allowing us to calculate optimal flow rates for microfluidic experiments specific to each patient. Notably, the Reynolds number for the human carotid artery is ≈200, indicating laminar flow with no turbulence. This allows for the safe downscaling of the vascular geometry to the microfluidic scale without significantly altering the overall flow pattern. Through iterative simulations and comparisons, we determined that a flow rate of 127 µL min^−1^ for patient #1‐H and 51 µL min^−1^ for patients #2 and #3 best matched the in vivo hemodynamic conditions.

A key finding from our CFD analyses was that peak shear rates for all patients exceeded 1000 s^−1^ (Figure 2, *4th column*, *magenta zone*), indicating pathological hemodynamic conditions. In comparison, the shear rate in healthy carotid arteries is ≈400 s^−1^.^[^ ^1^ ^]^ This observation aligns with the clinical understanding of disturbed flow patterns in stenotic vessels and provides valuable insights into the local hemodynamic patterns that may contribute to thrombosis risk.

Quantitative comparison of shear rates in the stenotic zone (black box region in the 4th column) between CTA and microfluidic scales showed no statistically significant differences (*p* > 0.05, ns), validating the geometric downscaling for experimental modeling (Figure 2, *5th column*). This similarity suggests that the microfluidic Carotid Artery‐Chip model accurately replicates the hemodynamics of the carotid artery and its branches for individual patients while preserving the anatomical accuracy of the vessels where flow disturbances occur, providing a robust platform for studying thrombotic events under physiologically relevant conditions.

### 2.3 Endothelialization and Biofunctionalization of the Carotid Artery‐Chip

To further enhance the physiological relevance of our Carotid Artery‐Chip, we implemented a comprehensive endothelialization and biofunctionalization protocol. This step was crucial for creating a model that not only replicated the physical structure of the carotid artery but also mimicked its biological properties.

**Figure 3:**
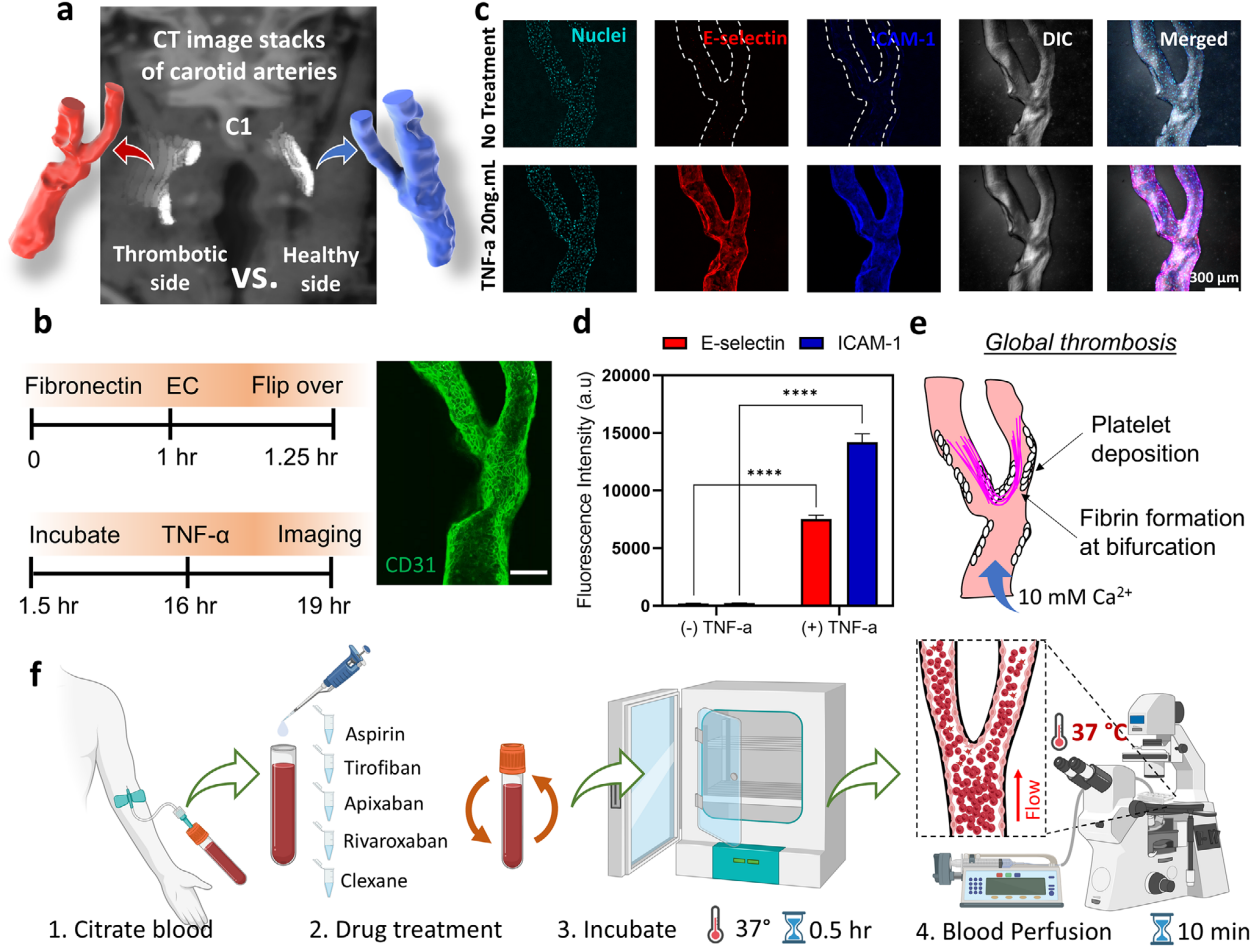
Endothelialization, induction of proinflammatory injury in Carotid Artery‐Chips, and blood perfusion procedure for thrombosis visualization. a) CTA image stacks of the left (thrombotic) and right (healthy) carotid arteries of patient #1‐H. The thrombotic side exhibits ≈70% stenosis in the ICA. b) *left*: Procedure for seeding endothelial cells in a bare Carotid Artery‐Chip, followed by treatment with tumor necrosis factor‐alpha (TNF‐α) to emulate an inflammatory state. *Right*: Representative confocal microscopy of CD31 (*green*) immunostaining confirms complete endothelialization for patient #1‐H's thrombotic side chip. Scale bar = 200 µm. c) Confocal microscopy and DIC imaging of functional endothelium lining within the Carotid Artery‐Chip for patient #1‐H's thrombotic side. Representative immunostaining images show the expression of E‐selectin (*red*) and ICAM‐1 (*blue*) with and without TNF‐α stimulation (*n* ≥ 3 independent experiments in duplicate). Nuclei staining (*cyan*) confirms complete endothelialization of the channel. Scale bar = 300 µm. d) Quantification of ICAM‐1 and E‐selectin fluorescent intensities in the presence (+) and absence (‐) of TNF‐α stimulation. Statistical significance is indicated by **** = *p *< 0.0001, assessed by unpaired, two‐tailed Student's t‐test with 50 iterations using an ROI randomizer. The data was obtained from *n* ≥ 3 patient #1‐H thrombotic side chips. e) Schematic representation of global thrombosis formation. Under high coagulation conditions stimulated by elevated calcium levels, fibrin‐rich blood clots are expected to form at the bifurcation area. f) Detailed procedure for blood perfusion starting from blood drawn from healthy donors with sodium citrate as an anticoagulant. The blood is treated with various drugs and incubated for 30 min before perfusion. Recalcification with specific amounts of calcium is performed immediately before the 10‐min perfusion experiments under 37 °C to achieve different experimental conditions.

Our patient cohort exhibited varying degrees of carotid artery stenosis, with patient #1‐H showing the most significant stenosis at the left ICA, while patient #2‐G presented with the least stenosis at the right ICA. Interestingly, neither of these two patients showed evidence of plaque rupture or endothelial injury in their carotid arteries at the time of CTA scanning. To investigate the underlying causes and potential thrombotic risks, we constructed and fabricated both left and right carotid arteries of patient #1‐H for comparison under physiological and hypercoagulable blood conditions (**Figure** **3**a). Notably, even on the right (healthy) side, the internal carotid artery exhibits a pathological stenotic region distal to the bifurcation.

The biofunctionalization process began with the preparation of microfluidic channels. We first coated the channels with fibronectin. Following this coating step, we seeded human umbilical vein endothelial cells within the microfluidic channels to emulate the human vessel wall (Figure 3b, *left*), which are widely utilized in vessel‐on‐chip settings for thrombosis studies.^[^ ^21^, ^22^, ^23^ ^]^

To ensure uniform endothelialization across the entire channel surface, we employed an established inversion technique. Fifteen minutes post‐seeding, the Carotid Artery‐Chip was carefully inverted, allowing the cells to adhere to all surfaces of the channel, including the upper walls. This approach resulted in a near‐complete and physiologically relevant endothelial lining compared to traditional static seeding methods (Figure S6, Supporting Information).

After overnight incubation, we observed the formation of a confluent endothelial cell monolayer, as confirmed by CD31 staining (Figure 3b, *right*). This confluent layer is critical for replicating the barrier function of the endothelium and its role in regulating thrombosis and inflammation.

To assess the biofunctionality of our endothelialized Carotid Artery‐Chip, we stimulated the endothelial cells with tumor necrosis factor‐alpha (TNF‐α), a proinflammatory cytokine. In mice, systemic TNF‐α robustly upregulates endothelial E‐selectin and intercellular adhesion molecule 1 (ICAM‐1), increasing leukocyte rolling and adhesion within hours.^[^ ^24^, ^25^ ^]^ Consistent with these reports, TNF‐α exposure in our Carotid Artery‐Chip increased E‐selectin and ICAM‐1 fluorescence across the endothelium (Figure 3c, Video S5, Supplementary Video5).

Quantitative analysis of fluorescence intensity revealed a significant upregulation of both ICAM‐1 and E‐selectin following TNF‐α stimulation (*p* < 0.0001, Figure 3d, data obtained from *n* ≥ 3 patient #1‐H thrombotic side chips). Specifically, we observed a 59.9‐fold increase in ICAM‐1 expression and a 36.0‐fold increase in E‐selectin expression. This robust response to inflammatory stimuli demonstrates the physiological relevance of our model and its potential for studying vascular inflammation in the context of thrombosis.^[^ ^26^ ^]^

### 2.4 Characterization and Modulation of Global Thrombosis in Patient‐Specific Carotid Artery‐Chips

**Figure 4:**
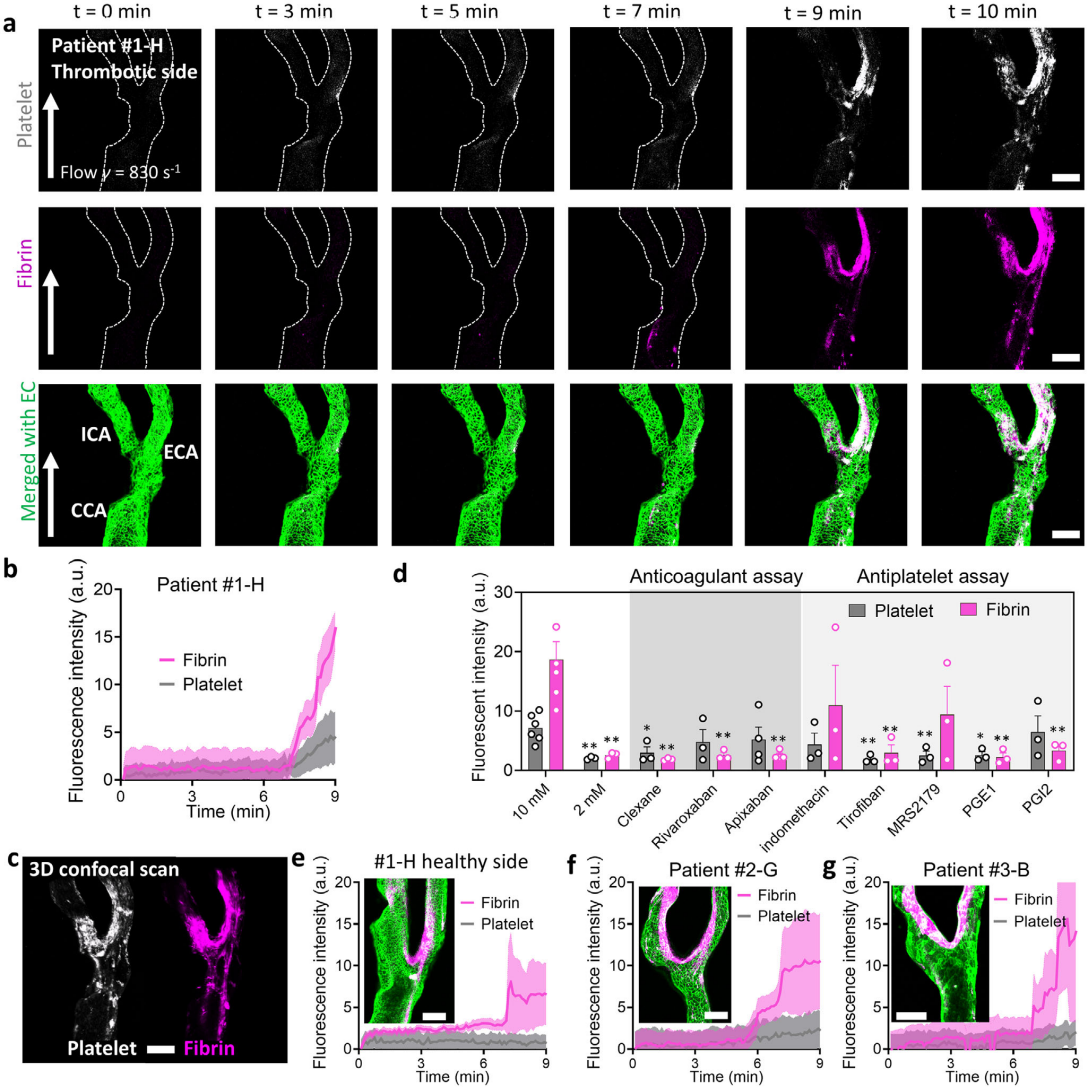
Blood perfusion for hypercoagulant tests, anticoagulant assay, and antiplatelet assay for comprehensive global thrombosis evaluation. a) Representative confocal images of patient #1‐H's Carotid Artery‐Chip under hypercoagulant blood perfusion, illustrating platelet aggregation (Row 1, *white*) and fibrin formation (Row 2, *magenta*) at the vessel bed (Row 3, *green*). The flow direction is from bottom to top, with platelet and fibrin signals captured at 0, 3, 5, 7, 9, and 10‐min time points under a 10 mM CaCl_2_ dose. Scale bar = 200 µm. b) Time‐lapse quantification of fibrin formation and platelet aggregation in patient #1‐H's Carotid Artery‐Chip over a 0 to 9‐min duration. The data was obtained from *n* ≥ 3 patient #1‐H thrombotic side chips. c) 3D confocal image stacks showing a representative image of platelet aggregation (*white*) and fibrin formation (*magenta*). d) Graph depicting fibrin formation and platelet aggregation after 10 mins perfusion within patient #1‐H's Carotid Artery‐Chip lumen when 2 mM and 10 mM recalcified citrate blood was perfused following the addition of anticoagulants, including clexane (5 U mL^−1^), rivaroxaban (500 nM), apixaban (500 nM); and antiplatelets, including indomethacin (10 µM), tirofiban (330 ng mL^−1^), MRS2179 (100 µM), prostaglandin E1 (PGE1; 1 µg mL^−1^), and prostacyclin (PGI2; 1 µg mL^−1^). The data was obtained from *n* ≥ 3 patient #1‐H thrombotic side chips. Statistical significance is indicated by * = *p* < 0.05, ** = *p* < 0.01, assessed by unpaired, two‐tailed Student's t‐test. e) Time‐lapse quantification and confocal images of fibrin formation and platelet aggregation for patient #1‐H's healthy carotid artery side, over a 0 to 9‐min duration. The data was obtained from *n* ≥ 3 patient #1‐H healthy side chips. f) Time‐lapse quantification of fibrin formation and platelet aggregation in patient #2‐G's Carotid Artery‐Chip over a 0 to 9‐min duration. The data was obtained from *n* ≥ 3 patient #2‐G thrombotic side chips. g) Time‐lapse quantification of fibrin formation and platelet aggregation in patient #3‐B's Carotid Artery‐Chip over a 0 to 9‐min duration. The data was obtained from *n* ≥ 3 patient #3‐B thrombotic side chips.

For patient #1‐H, who had 70% stenosis with no evidence of localized thrombosis through imaging, we focused our investigation on thrombotic response across the entire vasculature on a chip (Figure 3e). To simulate this “global” thrombotic condition and accelerate the thrombotic events, we perfused citrated human blood recalcified with 10 mM CaCl_2_ through the endothelialized Carotid Artery‐Chip at a calculated bulk shear rate of *γ*_0_ = 830 s^−1^ for 10 min (Figures 3f and 4a). The 10 mM concentration was selected based on prior in‐vitro thrombosis studies demonstrating robust yet controlled clot formation.^[^ ^26^ ^]^ This flow rate, determined by our earlier CFD analyses, ensured physiologically relevant hemodynamics. In addition, all blood perfusion experiments were conducted at 37 °C to maintain physiological coagulation system activity. We observed a time‐dependent increase in platelet and fibrin deposition within the microfluidic channel, with significant accumulation at the 10‐min mark. Merged images with endothelial cells (*green*) revealed that thrombosis predominantly occurred at the bifurcation of the Carotid Artery‐Chip (**Figure** **4**a).

Quantitative analysis of fluorescence signals for platelets and fibrin corroborated these qualitative observations, showing a marked increase in thrombus formation over time (Figure 4b, data obtained from *n* ≥ 3 patient #1‐H thrombotic side chips). Notably, the fluorescence intensity increased sharply after 7 min, indicating a critical time point in thrombus development. 3D confocal scanning provided insights into the spatial distribution of platelets and fibrin (Figure 4c). We observed that platelet adhesion occurred first, followed by fibrin deposition in regions of high platelet accumulation.

Leveraging the drug testing capability of our Carotid Artery‐Chip (Figure 3f), we evaluated the efficacy of various anticoagulant and antiplatelet drugs in reducing global thrombosis formation for patient #1‐H (Figure 4d; Video S6, Supplementary Video6). Compared to blood solely tested under recalcification, all three tested anticoagulants (clexane, rivaroxaban, and apixaban) significantly reduced fibrin formation. Clexane demonstrated the most potent effect, reducing both platelet aggregation and fibrin formation to levels close to the baseline set by blood recalcified with 2 mM CaCl_2_. Rivaroxaban and apixaban primarily inhibited fibrin formation.

Antiplatelet drugs exhibited diverse patterns of thrombosis inhibition. Indomethacin and MRS2179 moderately reduced platelet aggregation with a less pronounced effect on fibrin formation. In contrast, tirofiban, PGE1, and PGI2 effectively blocked both platelet aggregation and fibrin formation, demonstrating potent antithrombotic effects in this global thrombosis model.

To assess the impact of vascular health on thrombotic potential, we conducted similar experiments on the chip generated from the contralateral (healthy) carotid artery of patient #1‐H (Figure 4e, data obtained from *n* ≥ 3 patient #1‐H healthy side chips). This chip showed significantly lower levels of thrombus formation before 9 min, evidenced by reduced fluorescence intensity for both platelets and fibrin. This observation highlights the increased susceptibility to clot formation in the stenotic vessel, even in the absence of localized thrombosis visible through conventional imaging.

Extending our investigation to patient #2‐G and #3‐B, we observed comparable trends in global thrombus formation (Figure 4f, data obtained from *n* ≥ 3 patient #2‐G thrombotic side chips and 4g, data obtained from *n* ≥ 3 patient #3‐B thrombotic side chips). The fibrin deposition levels followed the expected pattern: #1‐H diseased (70% stenosis) > #2‐G (10% stenosis) > #1‐H healthy (0% stenosis), confirming the chip accurately reflects stenosis‐thrombosis relationships. The consistency across these patient samples underscores the reliability and potential clinical relevance of our Carotid Artery‐Chip platform, highlighting the potential impacts of stenotic geometries on the development of occlusive thrombi. The large error bar observed in patient #3‐B reflects biological variability, likely stemming from non‐stenotic factors such as local flow disturbances (Figure 4g). These findings collectively demonstrate the capability of our Carotid Artery‐Chip to reveal subtle differences in thrombotic tendencies between healthy and diseased vessel segments.

### 2.5 Integration of Laser Ablation Injury with Patient‐Specific Carotid Artery‐Chips for Localized Thrombosis Modeling

**Figure 5:**
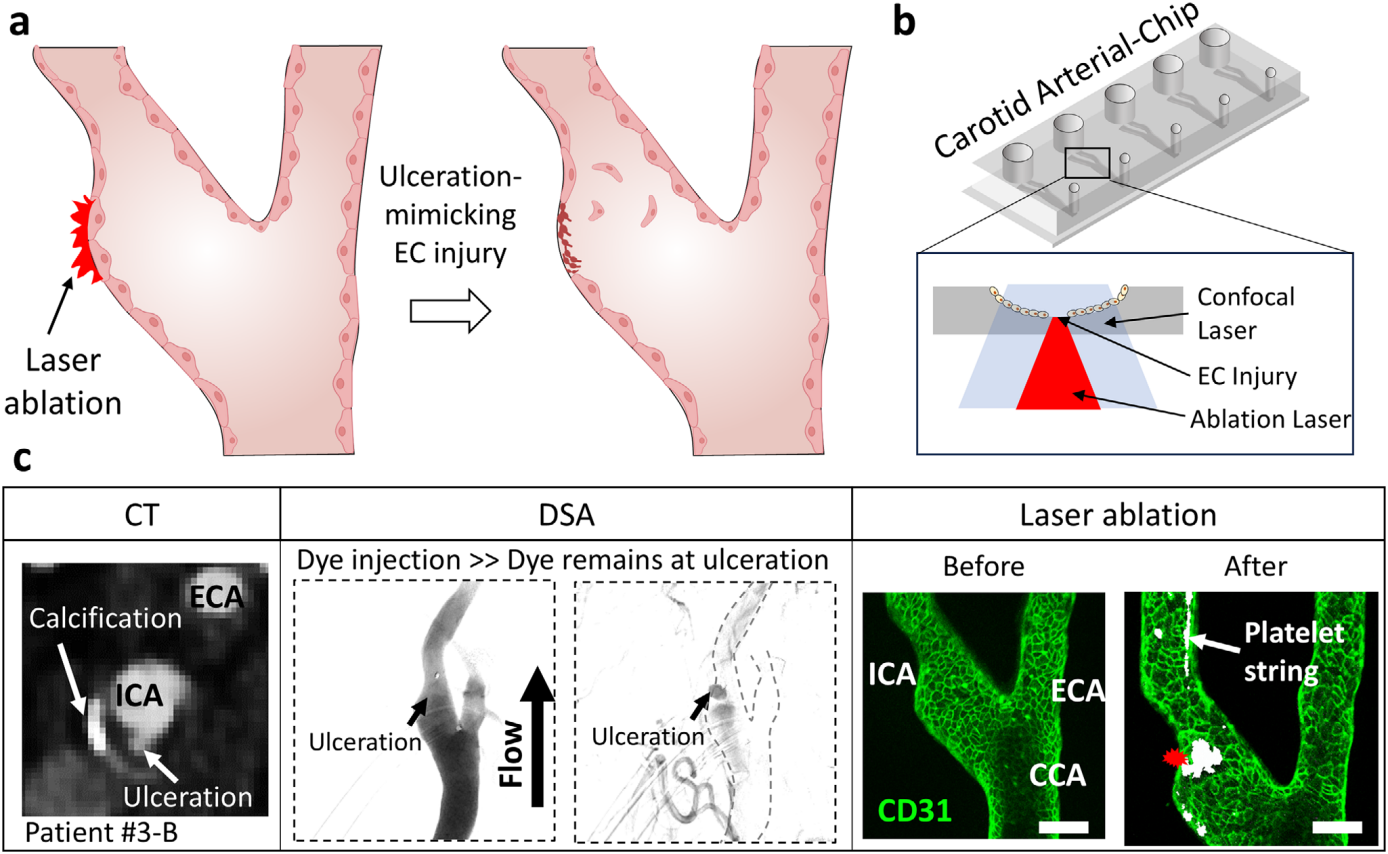
Process of creating endothelial cell injury using the laser ablation module on a Carotid Artery‐Chip. a) Schematic representation of endothelial cell injury via laser ablation. Plaque rupture causes endothelial injury, leading to platelet aggregation and localized thrombosis or embolization. b) The laser ablation process is shown on a Carotid Arterial‐Chip. The ablation site is calibrated and aligned according to actual patient information. A short pulse of laser is fired, causing endothelial cell injury. The inset illustrates the light path, including the confocal laser and ablation laser. c) Identification of vessel injury sites using clinical images. CTA Images (*1st column*) show calcification and potential ulceration sites in patient #3‐B. The suspected ulceration sites appear as abnormal bulbous zones within the vessel lumen. Dynamic blood flow images obtained via DSA Images (*2nd column*) confirm the ulceration sites by the persistence of dye in the vessel wall after the initial flush. Confocal microscopy images (*3rd column*) of CD31‐stained endothelial cells, indicating the sites of laser‐induced vessel injury within the Carotid Artery‐Chip (*left*). Scale bar = 200 µm. Zoom‐in images demonstrate the precise localization of distal and proximal injuries using a RAPP UGA‐42 Caliburn laser ablation module integrated with a confocal microscope (*right*). Platelet strings were observed after the laser ablation. Scale bar = 120 µm.

We developed an approach to study localized thrombosis by integrating laser‐induced injury with our patient‐specific Carotid Artery‐Chip platform (**Figure** **5**). This method adapts the laser injury technique, traditionally used in animal models,^[^ ^27^ ^]^ to a microfluidic environment with human cells and patient‐specific geometries. This setup allows for the induction of precise, controlled injuries at specific locations within the microfluidic channel. The process of creating endothelial cell injury using the laser ablation module involves targeted disruption of the endothelial layer to mimic plaque rupture (Figure 5a). In atherosclerotic disease, plaque rupture causes endothelial injury, exposing subendothelial components and leading to platelet aggregation and potentially thromboembolism. In our system, the laser ablation site is carefully calibrated and aligned according to actual patient information derived from clinical imaging (Figure 5b). A short pulse of laser is fired through the optical path alongside the confocal imaging beam, causing controlled endothelial cell injury while allowing simultaneous visualization of the thrombotic response. The extent of endothelial injury following laser ablation was consistently controlled to result in the loss of ≈5–7 cells, enabled by maintaining constant laser parameters (70% of maximum output power at 355 nm wavelength). Samples exhibiting cell loss outside this predefined range were excluded from analysis to ensure consistency and reproducibility.

We employed a multi‐modal imaging approach to identify vessel injury sites in patients. CTA images were used to locate fibrocalcification and potential ulceration sites in the carotid arteries of patient #3‐B, which appeared as abnormal bulbous zones within the vessel lumen (Figure 5c, *1st column*). DSA further refined the localization by revealing areas of blood penetration into the vessel wall, evidenced by the persistence of dye after the initial flush (Figure 5c, *2nd column*).

Translating these clinical observations onto our Carotid Artery‐Chip, we used confocal microscopy to visualize CD31‐stained endothelial cells and target our laser ablation (Figure 5c, *3rd column, left*). This approach allowed us to create injuries that mimic those observed in patients. Following laser ablation, we observed the formation of platelet strings at the injury sites (Figure 5c, *3rd column, right*). This immediate thrombotic response, visualized in real‐time, demonstrates the physiological relevance of our model, offering insights into the early stages of thrombus formation and the critical influence of endothelial injury.

**Figure 6:**
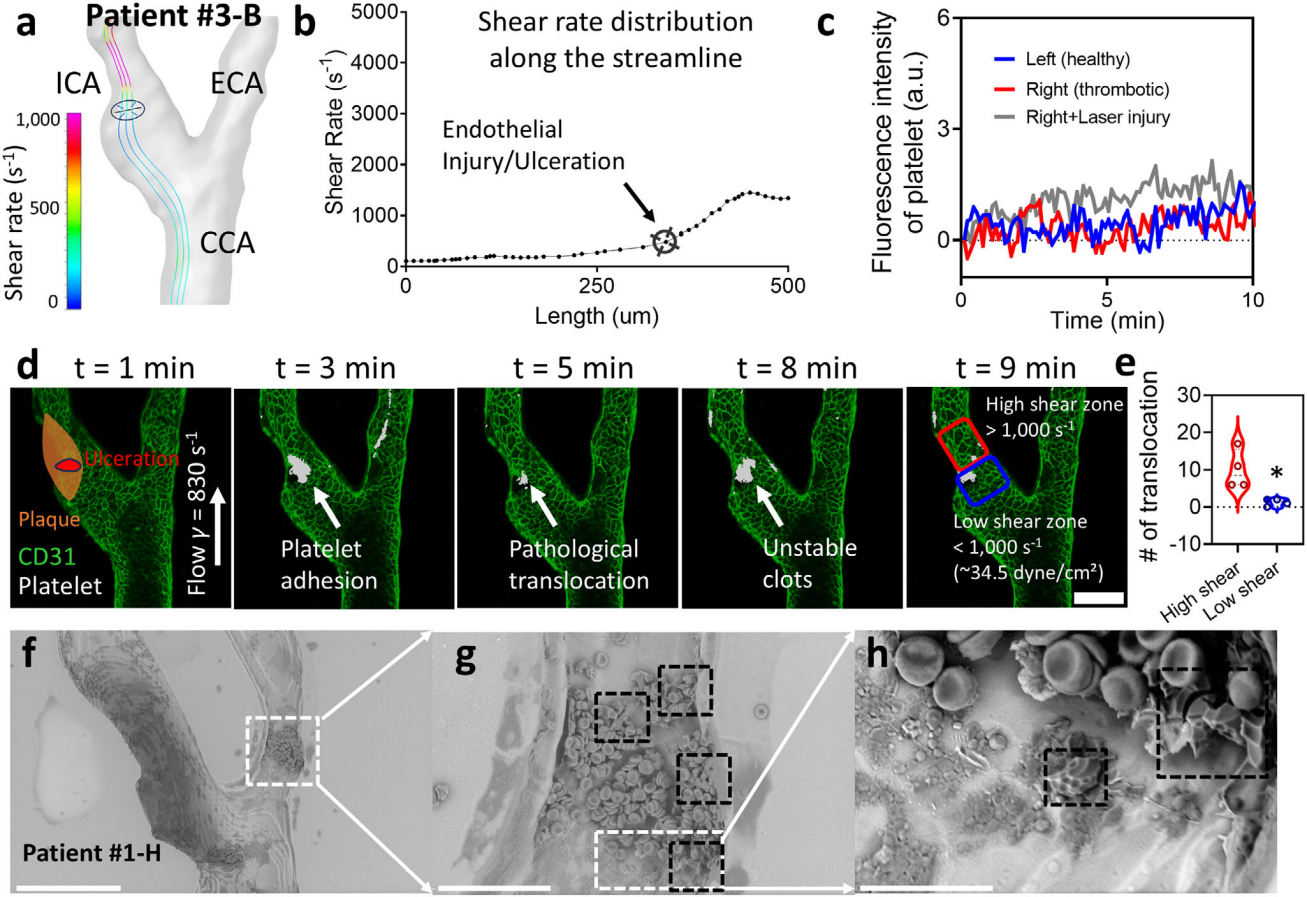
Assessment of localized thrombosis in patient #3‐B. a) Visualization of the shear rate distribution along the carotid artery bifurcation at the microfluidic scale. The crosshair highlights the region of endothelial injury/ulceration in patient #3‐B, which is also the laser ablation location. The streamlines indicate elevated shear rates at the site of injury. b) Line graph demonstrating the shear rate along the length of the streamline. The area of endothelial injury/ulceration corresponds to a significant increase in shear rate. c) Time‐lapse fluorescence intensity of platelets under physiological conditions for the healthy (*blue*) and thrombotic (*red*) carotid artery sides of patient #3‐B, and the laser injury condition for the thrombotic side (*gray*) over a 10‐min period. d) Representative confocal images of platelet adhesion, aggregation, and translocation in the endothelialized Carotid Artery‐Chip of patient #3‐B at various time points (1, 3, 5, 8, and 9 min). Note that platelets accumulate and are translocated multiple times at the ulceration site. The flow direction is from bottom to top, with a bulk shear rate of γ = 830 s^−1^. Scale bar = 200 µm. e) Quantification of platelet translocation events in high shear (> 1000 s^−1^; ∼ 34.5 dyne cm^−2^, red) versus low shear (< 1000 s^−1^, blue) regions of equal area. Data show a ≈7–10‐fold higher translocation density in the high shear zone. Statistical significance is indicated by * = *p* < 0.05, assessed by unpaired, two‐tailed Student's t‐test. The data was obtained from *n *≥ 3 patient #3‐B thrombotic side chips. f‐h) Fixed (paraformaldehyde, 4%) blood clots on endothelial cells formed within the microfluidic devices representing Patient #1‐H were observed with SEM at f) 640x (Scale Bar = 200 µm), g) 2800x (Scale Bar = 50 µm), and h) 11000x magnification (Scale Bar = 15 µm). White dashed box: zoomed in ROI. Black dashed box: regions of polyhedrocytes.

To investigate the effect of hemodynamic conditions on thrombus formation, we focused on the Carotid Artery‐Chip made for patient #3‐B, explicitly examining the region of endothelial injury identified by CTA and DSA. We first utilized CFD simulation to examine shear rate distribution and reveal local hemodynamics at the site of ulceration identified in patient #3‐B's carotid artery. The simulation showed an increase in shear rate along the flow streamline at the site of endothelial injury (**Figure** **6**a,b). To establish a baseline, we tested platelet activity under physiological conditions using Carotid Artery‐Chips molded from both the thrombotic (*Right*) and contralateral healthy (*Left*) carotid arteries (Figure 6c). Although no significant differences were observed in platelet signal magnitudes across these conditions, the dynamic process revealed notable variations, with an increased frequency of translocation events observed under the laser injury condition (Video S7, Supplementary Video7).

Real‐time confocal scanning allowed us to investigate the dynamic process of platelet adhesion, aggregation, and translocation at the ulceration site (Figure 6d). We observed rapid platelet accumulation and multiple translocation events within the high shear environment, highlighting this region's susceptibility to thrombus formation and thromboemboli release. This behavior may be attributed to the shear environment distribution in the carotid artery, where platelet aggregates forming in higher shear rate areas (Figure 6a, *purple streamlines*) may detach and potentially occlude downstream vessels.

To quantify the shear dependence of platelet translocation, CFD shear maps were co‐registered to the imaging field, and equal‐area region of interests were defined for high shear zone (>1000 s^−1^; Figure 6d red rectangular) and low shear zone (<1000 s^−1^; Figure 6d blue rectangular). Translocation event was ≈7–10‐fold higher in the high shear zone (median ≈9) than in the low shear zone (median ≈1), indicating shear rate‐related translocation events (Figure 6e, data obtained from *n* ≥ 3 patient #3‐B thrombotic side chips).

Last but not least, to further characterize the structural features of blood clots formed within our microfluidic devices, we performed scanning electron microscopy (SEM) on fixed thrombi formed in the chip representing Patient #1‐H (Figure 6f–h). At lower magnification (640x), we observed the overall architecture of the clot forming on the endothelial surface (Figure 6f). Higher magnification (2800x) revealed the intricate network of platelets and fibrin forming the structural scaffold of the thrombus (Figure 6g). At the highest magnification (11000x), we could distinguish individual cellular components, including polyhedrocytes – polyhedral erythrocytes compressed within the clot structure (Figure 6h, black dashed box), which are characteristic features of mature thrombi. These SEM images confirm that the thrombi formed in our microfluidic devices recapitulate the key structural elements observed in pathological thrombi in vivo.^[^ ^28^ ^]^

## 3 Discussion

In this study, we introduced a novel approach to rapidly microfabricate patient‐specific Carotid Artery‐Chips based on CTA images from stroke patients, offering a dynamic and more accurate to‐date model for cerebrovascular thrombosis that addresses key limitations of current static image‐based diagnostic methods. Our findings classified thrombotic events into global and localized thrombosis, providing a more nuanced understanding of the thrombotic process.

The use of treated glass slides as printing substrates represents a crucial innovation, eliminating the need for bulky bases and dramatically reducing printing time from over 10 h to ≈2 h. This modification, combined with our custom locator system for precise substrate positioning, ensures consistent high‐fidelity reproduction of intricate vascular features while enhancing manufacturing efficiency. The dual‐material clamping approach further resolves the common challenge of leakage in multilayer microfluidic devices, providing a robust solution for maintaining structural integrity during experimental procedures.

A distinguishing feature of our approach is the meticulous preservation of patient‐specific anatomical details through manual refinement of automated reconstructions. Initial vessel reconstructions were performed using automated algorithms, but in cases where high‐contrast calcified plaque or ulcerations obscured the lumen boundary, manual refinements were undertaken in consultation with neurologists on the team. These refinements involved evaluating upstream and downstream vessel geometry for anatomical continuity and enhancing local contrast to confirm vessel wall integrity. Our findings reveal that conventional automated methods often fail to accurately capture critical features such as ulcerations and complex stenoses, which are precisely the most relevant regions for thrombotic risk assessment. This attention to anatomical fidelity is essential for studying the relationship between vessel geometry and thrombotic tendency, as demonstrated by our observation of differential thrombotic responses between stenosed and contralateral vessels from the same patient.

The laser ablation protocol recreates the proximal trigger for thrombotic stroke–plaque rupture and endothelial injury. By localizing ablation to patient‐specific ulceration sites identified via CTA/DSA and combining with individualized flow conditions, we can assess “what‐if” scenarios of plaque rupture that are otherwise untestable in vivo. This approach reveals patient‐specific susceptibility to thromboembolism following rupture events, filling a critical gap in current risk stratification, which relies primarily on anatomical measurements.

Moreover, our study revealed that the translocation of aggregated platelets in localized thrombosis is influenced by shear rate distribution. Platelet aggregation initially occurred in a low shear zone (<1000 s^−1^) and grew into the high shear zones (>1000 s^−1^), where translocation is likely initiated. Quantitative analysis demonstrated a ≈7–10‐fold higher translocation density in the high shear zone (>1000 s^−1^), providing mechanistic insight into shear‐dependent embolization risk. These findings highlight the critical role of shear forces in the dynamics of platelet thrombus formation and progression and its detachment to become thromboemboli, underscoring the need for therapies that effectively regulate and target different stages of shear‐induced platelet behaviors.

The observed translocation events at sites of endothelial injury highlight a potentially important mechanism in thromboembolism, suggesting that regions of altered hemodynamics, even in vessels with stenosis levels below clinical intervention thresholds, may harbor significant thrombotic risk. This finding could have important implications for understanding why some patients with apparently “low‐risk” stenosis still develop strokes.

While our Carotid Artery‐Chip platform offers several advantages, it is essential to consider its limitations and the need for further development. The system currently focuses on the carotid artery and may not fully represent thrombotic processes in other vascular beds. Additionally, while we have incorporated endothelial cells and platelets, the model does not yet account for all blood and vessel wall components that may influence thrombosis. Future iterations could benefit from including additional cell types, such as smooth muscle cells and leukocytes, to create an even more comprehensive model of the vascular environment. While preserving wall shear rate maintains the primary mechanical stimulus for endothelial mechanotransduction, platelet adhesion, and aggregation,^[^ ^9^, ^29^ ^]^ we cannot simultaneously preserve Reynolds number, Womersley number, and wall shear rate. Time‐varying pulsatile effects and inertial forces are not fully captured at the microscale. However, wall shear stress – the key driver of thrombotic biology – is accurately preserved, ensuring biological relevance despite geometric downscaling.^[^ ^30^ ^]^ In addition, the applied flow conditions were based on literature‐derived averages and could be further refined through the incorporation of direct patient‐specific flow measurements.

In conclusion, our patient‐specific Carotid Artery‐Chip platform represents a significant step forward in the dynamic modeling of cerebrovascular thrombosis, offering a more comprehensive view of the thrombotic process and enabling personalized evaluation of various antithrombotic strategies.^[^ ^31^ ^]^ However, it should be regarded as a powerful research tool and potential aid to clinical decision‐making, rather than a standalone diagnostic or treatment planning device.

## 4 Experimental Section

### Reagents and Cell Lines

Human umbilical vein endothelial cells were purchased from Lonza Bioscience, #C2519A and cultured in EGM‐2 BulletKit medium (Lonza Bioscience, #CC‐3162) until 80%–90% confluent. Anti‐CD31 Alexa Fluor 488 (Clone JC/70A, Ab215911) was prepared at a 1:200 dilution in EGM‐2 medium. CD41 freeze‐dried antibody (P2) was obtained from Beckman Coulter, #IM0145, and reconstituted and labeled with Atto 488 NHS Ester (Sigma Aldrich, #41698‐1MG‐F) to 0.8 mg mL^−1^. Anti‐human fibrin antibody, hybridoma clone 59D8, was from Gary Matsueda, Harvard University, USA, labeled with Alexa 647 NHS Ester (Invitrogen, #A37573) to 0.75 mg mL^−1^. The primary E‐selectin Monoclonal Antibody (1.2B6) was obtained from Invitrogen, #MA122165, 1 mg mL^−1,^ and used at 2ug mL^−1^; Secondary Alexa Fluor 555 conjugated goat anti‐human secondary was obtained from Invitrogen, #A‐21433, 2 mg mL^−1,^ and used at 2ug mL^−1^. Hoechst 33342 was obtained from Sigma Aldrich, #14533‐100MG, and used at 1:4000 dilution in PBS. Anti‐human ICAM‐1 (HA58) was obtained from Invitrogen, #17‐0549‐42, 100 ug mL^−1,^ and used at 5 ug mL^−1^. TNFα was obtained from Stem Cell Technologies, #78068, and diluted to 20 ng mL^−1^ in EGM‐2 medium.

After collecting blood from donors, it is treated with various drugs and incubated for 30 min before perfusion.^[^ ^32^ ^]^ Recalcification with specific amounts of calcium is performed immediately before the 10‐min perfusion experiments under 37 °C to achieve different experimental conditions. Rivaroxaban was obtained from Sigma Aldrich, #SML2844‐5MG, and reconstituted in DMSO to 4.59 µM. Apixaban was obtained from Cayman Chemical, #15427, and reconstituted in DMSO to 10.88 mM. Tirofiban was obtained from Sigma Aldrich, #30165, and reconstituted in saline to 0.25 mg mL^−1^. Clexane was obtained from Sanofi Medical, 100 mg/mL (10 000U). Prostaglandin E1 and Prostacyclin were obtained from Sigma Aldrich. Indomethacin was obtained from ChemSupply Australia #GA3090‐25G, and reconstituted in DMSO to 20 mM. MRS2179 was obtained from Sapphire Bioscience, #10011450‐10MG, and reconstituted in DMSO to 100 mM.

### Patient Selection and Clinical Data Collection

Three patients were selected for this study based on documented cases of carotid artery‐related stroke at Royal Prince Alfred Hospital (RPAH). Totally five carotid artery geometries were extracted from these patients, including left and right C1 segments for Patient #1‐H and #3‐B, and a stenosed right carotid artery for Patient #2‐G. The inclusion criteria were based on the availability of high‐quality CTA and DSA imaging data (New South Wales Telestroke Service),^[^ ^33^ ^]^ essential for accurate 3D reconstruction and hemodynamic analysis. Patients with severe systemic diseases, coagulation disorders, or previous carotid artery interventions were excluded to ensure a homogeneous study population.

Clinical data were collected in accordance with ethical guidelines and patient consent protocols approved by the Sydney Local Health District Human Research Ethics Committee (HREC)–RPAH Zone (X23‐0267 & 2023/ETH01607). The collected data included demographic information (age, gender, and relevant medical history) as well as high‐resolution CTA images for each patient.

Patient #1‐H (67 years old) presented with an acute left hemispheric stroke, two months after experiencing a transient ischemic attack in the same hemisphere. CTA confirmed fibrocalcific plaque with ≈70% stenosis at the left C1 segment. Patient #2‐G (71 years old) presented with right hemispheric symptoms of facial asymmetry, dysphasia, dysphagia, and dysarthria. CTA revealed ≈10% stenosis at the right C1 segment without calcification or ulceration. Patient #3‐B (78 years old) presented with an acute right hemispheric stroke. CTA and DSA confirmed fibrocalcific plaque with ulceration and ≈40% stenosis at the right C1 segment.

### Acquisition and Processing of Clinical CTA Images

High‐resolution CTA images were obtained for each patient to ensure accurate 3D reconstruction and analysis of carotid artery geometries. The imaging protocols were standardized across all patients to maintain consistency and quality. CTA scans were performed using a General Electric Optima CT660 CT scanner. The images were acquired with a slice thickness of 0.625 mm and pixel spacing of 0.539×0.539 mm, providing detailed visualization of the carotid artery structures. An 80 mL bolus of iodinated contrast was administered intravenously to enhance the visibility of the vascular lumen and detect any stenotic or ulcerative lesion (4–7 mL s^−1^), followed by a saline flush. All images were transmitted to a central repository, accessed by clinicians at the time of the consult.

The acquired CTA images underwent processing to prepare them for 3D reconstruction and analysis. Scans with motion artifacts or insufficient contrast were excluded. The reviewed images were imported into SimVascular, an open‐source software developed by the Cardiovascular Biomechanics Computation Lab at Stanford University.^[^ ^34^ ^]^ Initial vessel reconstructions were performed in SimVascular using the 3D region‐growing algorithm. In cases where high‐contrast calcified plaque or ulcerations obscured the lumen boundary, manual refinements were undertaken in consultation with neurologists on the team. Ulcerations were distinguished from the true lumen by evaluating the upstream and downstream vessel geometry for anatomical continuity, and by enhancing local contrast to confirm that the vessel wall remained continuous without abrupt angles. These refinements were performed using the 2D trimming tool in SimVascular, applied slice‐by‐slice to the CTA dataset to delineate the true lumen. The 3D models were exported as STL files and imported into SpaceClaim software (ANSYS Inc., 2023) for additional surface refinements and preparation for microfluidic fabrication as previously described.^[^ ^18^, ^19^ ^]^

### Fabrication of Patient‐Specific Carotid Artery‐Chips—Preparation of the Design File for Printing

The reconstructed vessel was digitally split into two halves along the middle axial plane (Figure 1b, *left*). Each half vessel was duplicated five times and separated by 15 mm, evenly distributed along the longer direction of the glass slide (Figure 1b, *left*). The CCA, ICA, and ECA were extended using cylindrical tubes to form microfluidic channels. The tube diameter, angle, and area of intersection were carefully refined to ensure smooth flow. Four spacers with a height of 230 µm were integrated into the bottom vessel design to precisely control the separation between the mold and the casting glass slide. The spacer shape was modified from cubic to hemispherical to prevent detachment from the glass slide substrate (Figure S3a,b, Supporting Information). The final designs were sliced using Voxeldance Additive software for 3D printing, with a layer thickness of 5 µm, represented as 8‐bit pixel depth PNG images.

### Pre‐Printing Preparation

Surface Treatment of Glass Slides. The silanization process for the glass slides involved the following steps (Figure S1, Supporting Information):

1. Sonication in an isopropanol bath for 5 min.
2. Blow‐drying with compressed air and sonication in Milli‐Q water for 5 min.
3. Soaking in a solution of Toluene anhydrous (99.8%) and 3‐(Trimethoxysilyl) propyl methacrylate (TMSPM, 98%) in a 90/10 w/w ratio for 2 h.
4. Rinsing with pure ethanol and blow‐drying with compressed nitrogen. All chemicals were procured from Sigma‐Aldrich (Toluene: 244511, TMSPM: 440159).

A custom locator comprising four L‐shaped brackets was integrated into the design to ensure that the vessel was printed at the center of the glass slide (Figure S4, Supporting Information). The glass slide was accurately placed inside the L‐shaped brackets and secured on the build platform by curing three tiny drops of printing resin between the glass slide and the build platform (Video S1, Supplementary Video1).

### 4.1 Printing Process

The BMF S240 DLP 3D printer was configured to print in stitch mode to achieve a total printing area of 100 × 100 mm^2^. The printing area was manually adjusted to avoid any intersection between the vessel geometry and the stitch lines caused by this mode. During the printing process, distinct printing parameters were set for each printing layer. For the first layer, which touches the glass substrate, an exposure time of 1.5 s with an exposure intensity of 60 mW cm^−^^2^ was used to achieve optimal adhesion. The exposure intensity was reduced to 45 mW cm^−^^2^ for the subsequent four layers to balance detail retention and layer adhesion. From the fifth layer to the sixth from the last layer, the exposure time was reduced to 1 s while maintaining the same exposure intensity to maximize detail recovery. For the final five layers, the exposure time was increased to 1.5 s to ensure the successful printing of ultra‐fine details. The height of each layer was controlled by adjusting the building platform's dropping and rising distance between each layer. After completing one layer, the build platform descended by 4 mm to release the cured resin from the release film, followed by a 3.995 mm ascent, forming a 5 µm resin layer for the next layer printing. Additionally, a 300‐s waiting time between layers was mandatory to ensure uniform layer thickness.

### Post‐Printing Process

The glass slide was carefully peeled off the build platform, washed in 100% ethanol, and post‐cured using a Form Cure machine (Formlabs) at 50 °C for 60 min (Video S2, Supplementary Video2). The molds were then silanized with Trichloro(1H,1H,2H,2H‐perfluorooctyl) silane (Sigma‐Aldrich: 448931) via vacuum vapor deposition as described previously.^[^ ^18^, ^19^ ^]^

### Microfluidic Chip Fabrication

PDMS (Sylgard 184, Dow Corning) was prepared at 10:1 w/w ratio between the base material and the curing agent. For the top vessel PDMS chip, an aluminum frame was used to standardize the chip's shape and thickness (Figure S5, Supporting Information). The PDMS mixture was poured into this frame, degassed, and cured for 2 h at 70 °C in an oven. Inlets (6 mm) and outlets (1 mm) were punched to accommodate flow experiments (Video S3, Supplementary Video3). A mechanical clamp system was designed using a Formlabs 3D printer. The upper clamp was made with Formlabs Rigid 4k resin, and the lower clamp with Formlabs Rigid 10k resin (Figure 1b, *right*). After clamping, the chip was sterilized using 80% ethanol and dried with nitrogen gas (Video S4, Supplementary Video4).

### Endothelialization and Inflammatory Stimulation of Carotid Artery‐Chips

Human umbilical vein endothelial cells (passages 6–10) were resuspended using TrypLE Express Enzyme (#12604021, Thermo Fisher Scientific) at 5×10^6^ cells mL^−1^. Carotid Artery‐Chips were sterilized with 80% ethanol, washed with DPBS, and coated with 100 µg mL^−1^ human plasma fibronectin (#33016015, Thermo Fisher Scientific) for 1 h at 37 °C. After washing, 8 µL of endothelial cell suspension was injected into each channel through the outlet port (Video S8, Supplementary Video8). The excess suspension was removed from the big inlet to prevent the cell from flowing out of the channel. Chips were flipped and incubated for 15–20 min to allow cell attachment to the top half of the lumen, then the chips were flipped again for bottom lumen coverage. Chips were incubated overnight with EGM‐2 medium for complete endothelialization. To simulate inflammatory conditions, the endothelialized chips were incubated with 20 ng mL^−1^ Tumor Necrosis Factor Alpha for 4 h.

To visualize the endothelial monolayer prior to blood experiments, cells were incubated with EGM medium containing 2 ug mL^−1^ CD31‐488 antibody for 30 min in the incubator (see section 4.1). For immunostaining, chips were fixed in 4% paraformaldehyde for 15 min, washed, and blocked with 5% bovine serum albumin (BSA) for 1 h. Primary E‐selectin antibody was incubated overnight in 2% BSA at four degrees. Secondary 555 antibody (1 h) and ICAM‐1 (2 h) were added afterward with thorough washing in between steps. Hoechst was added last prior to imaging (10 min).

### Blood Perfusion on a Carotid Artery‐Chip

Blood collection from healthy donors was approved by the University of Sydney Human Research Ethics Committee (HREC, project 2023/HE000582). Donors provided written informed consent and were screened for appropriate age, weight, and absence of anticoagulant or anti‐inflammatory medication use. Blood was collected via a 19G butterfly needle into a syringe containing 3.8% sodium citrate anticoagulant. Whole blood was stained with anti‐CD41 and anti‐fibrin antibodies.

### Real‐Time Thrombosis Visualization and Quantification

An Olympus FV3000RS confocal microscope with a 10× UPlanXApo objective was used for real‐time imaging. The focal plane was set 30 µm above the bottom of the vessel lumen. For 2D real‐time imaging, platelet and fibrin fluorescence signals were acquired using the default pinhole settings with 550 and 647 nm excitation wavelengths, respectively. The receiving wavelengths corresponding to 405, 488, 550, and 647 nm excitation were 430–480, 500–550, 565–610, and 660–700 nm, respectively. To enable comprehensive visualization of the endothelial layer, CD31 signals were captured using a widened 800 µm pinhole with 488 nm excitation, allowing deeper optical sectioning. 3D z‐stack imaging was performed with a step size of 4 µm, capturing 50–70 slices depending on the depth of the patient‐specific Carotid Artery‐Chip geometry. For cellular staining, nuclei were visualized using 405 nm excitation, VE‐cadherin with 488 nm, and F‐actin with 647 nm. Quantitative analysis of fluorescently labeled platelets and fibrin was performed frame‐by‐frame using IMARIS software (Bitplane AG, Oxford Instruments).

### Laser Ablation Induced Endothelial Injury on a Chip

To study localized thrombosis, we integrated a laser injury model with our Carotid Artery‐Chip platform. A RAPP UGA‐42 Caliburn laser ablation module was coupled with the confocal microscope system. This setup allowed for precise, controlled endothelial injury at specific locations within the microfluidic channel, mimicking plaque rupture or endothelial damage. Injury sites were selected based on patient‐specific CTA and DSA imaging data, focusing on areas of potential vulnerability such as bifurcations or regions of altered shear stress. The laser parameters (power, duration, and spot size) were optimized to induce localized endothelial damage without compromising the overall structure of the microfluidic channel. Laser ablation was performed with a UGA‐42 Caliburn 355/42 (355 nm pulsed DPSS; 42 µJ pulse^−1^ at 1 kHz; average output 42 mW; Rapp OptoElectronic, Wedel, Germany). Each ablation was performed at 70% of the maximum output power. The laser was guided to trace a linear path along the identified ulceration site, maintaining a consistent ablation length across all samples. The extent of endothelial injury following laser ablation was consistently controlled to result in the loss of ≈5–7 cells, enabled by maintaining constant laser parameters. Samples exhibiting cell loss outside this predefined range were excluded from analysis to ensure consistency and reproducibility. The chips were immediately perfused with recalcified blood following laser ablation to simulate patient‐specific flow conditions. Post‐injury, platelet aggregates and potential embolization formation were monitored in real‐time using the confocal microscopy setup described in previous section.

### Statistical Analysis

All experiments were performed in triplicate unless otherwise stated. Data are presented as mean ± standard error of the mean (s.e.m.). Statistical significance was determined using unpaired Student's t‐tests for comparisons between two groups. *p*‐values < 0.05 were considered statistically significant. All statistical analyses were performed using GraphPad Prism 9 software.

### Scanning Electron Microscopy

SEM was performed using a benchtop Phenom XL scanning electron microscope (Thermofisher Scientific, USA), on dried and fixed non‐gold‐coated samples. Images were taken at 600–11000× magnification under low vacuum conditions (60 Pa) using a full back scatter detector configured with a working distance of ≈3.2 mm at 5 keV.

## Conflict of Interest

The authors declare that they have three patents related to the technology described in this article, as follows: 1) J.L.A., A.T., P.F., Z.Y.C., W. Z. (2025). “Microprecision 3D Printing for Personalized Ischemic Stroke Risk Assessment and Antithrombotic Drug Screening”, Australia Patent 2025900592. 2) Z.Y.C., J.L.A., W.Z., Z.Y. (2024). “A 3D printing patient‐specific microvascular fabrication methodology.” PCT/AU2024/050185; Australia Patent 2023900588. 3) Z.Y.C., J.L.A., W.Z. (2024). “Mechanical Clip System for Reversible, Leak‐Free Assembly of Microfluidic Device.” PCT/AU2024/051191; Australia Patent 2023903706. This potential conflict of interest has been disclosed and managed in accordance with the journal's policy on the declaration of conflicting interests.

## Author Contributions

L.A.J. and Y.C.Z. designed the research, analyzed data, and wrote the manuscript. Y.C.Z designed the chip, performed blood perfusion, and conducted analysis and interpretation of data. Z.W. designed the chip, developed the 3D printing method, refined the PDMS fabrication method, made the chips, and performed data analysis. A.N. co‐designed the chip and performed immunostaining and data analysis. A.S. performed the CFD simulations, SEM imaging, and data analysis. Z.W., Y.Z., and J.R. helped culture the endothelial cells and functionalize the chips. H.Z. and N.A.Y. performed data analysis and wrote the manuscript. Z.L., K.S.B., and F.P. provided clinical suggestions, co‐wrote, and revised the paper. T.A. provided the CTA images and contributed the reconstruction. L.A.J. is the senior and corresponding author.

## Supporting information



Supporting Information

Supplementary Video1

Supplementary Video2

Supplementary Video3

Supplementary Video4

Supplementary Video5

Supplementary Video6

Supplementary Video7

Supplementary Video8

## Data Availability

All experiments were performed in accordance with relevant guidelines and approved by the University of Sydney Human Research Ethics Committee. Informed consent was obtained from human participants of this study. All data that support the findings of this study are available on request from the corresponding author.
