# Supplementary material for: Rapid Glass‐Substrate Digital Light 3D Printing Enables Anatomically Accurate Stroke Patient‐Specific Carotid Artery‐on‐Chips for Personalized Thrombosis Investigation
Source: Adv Mater. 2025 Sep 11;38(5):e08890. doi: 10.1002/adma.202508890 (PMC12822522; doi:10.1002/adma.202508890)
Supplement: Supplementary file 1 — Supporting Information [file ADMA-38-e08890-s006.pdf]

ADVANCED MATERIALS

Supporting Information

for *Adv. Mater.*, DOI 10.1002/adma.202508890

Rapid Glass-Substrate Digital Light 3D Printing Enables Anatomically Accurate Stroke Patient-Specific Carotid Artery-on-Chips for Personalized Thrombosis Investigation

*Yunduo Charles Zhao, Zihao Wang, Arian Nasser, Allan Sun, Zhao Wang, Yingqi Zhang, Jianfang Ren, Haimei Zhao, Nicole Alexis Yap, Yinyan Wang, Zhiyong Li, Ken S Butcher, Freda Passam, Timothy Ang and Lining Arnold Ju**

Supplementary Methods

Computational Fluid Dynamics (CFD) Analysis

To ensure physiologically relevant hemodynamic conditions in our Carotid Artery-Chips, we performed comprehensive CFD simulations using ANSYS Fluent 2020 R1 software (version 20.1; Canonsburg, PA, USA) as aforementioned[1, 2]. Patient-specific 3D geometries, derived from the STL files used for chip fabrication, were imported into the ANSYS environment for preprocessing and mesh generation.

Mesh Generation: A tetrahedral dominant mesh method was employed across the geometry with a maximum element size of 50 μm. To capture the complex flow dynamics at the bifurcation, a sphere of influence centered at this region and spanning the width of the domain was applied with a refined element size of 5 μm. An inflation layer with smooth transitions was applied to the geometry, with the vessel walls acting as inflation boundaries. Default growth rate values of 1.2 were used, with a maximum of 5 layers applied. This approach ensured accurate resolution of boundary layer effects while maintaining computational efficiency.

Fluid Properties and Boundary Conditions: Blood flow through the geometry was assumed to be laminar and steady, with Newtonian and constant fluid properties. The density was set at 1060 kg/m^3, and the viscosity was set at 0.00345 (Pa s). A velocity inlet was chosen representing a constant wall shear rate of $415 s^{-1}$ at the inlet, which matches similar values as indicated from the literature representing the average in vivo wall shear rates produced in the carotid artery measured clinically[3, 4]. A zero-pressure outlet was used for all simulations for the geometries following vessel bifurcation at the internal carotid artery (ICA) and external carotid artery (ECA). No-slip conditions were applied at the walls, which were assumed to be rigid for this analysis. Under normal physiological conditions, the Reynolds number (Re) in the human carotid artery is typically within the laminar regime. A 1994 radiological review documented an average Re of ~200[5], and subsequent studies combining Doppler ultrasound and CFD have confirmed a mean Re ≈ 266 and peak systolic Re ≈ 911[6]. In our study, Re was derived from measured inlet velocities and vessel diameters for both in vitro and in vivo conditions, yielding Re ≈ 3.0 and Re ≈ 670, respectively, well below the conventional turbulent threshold of ~2000. Even in severely stenotic carotid arteries (>80%), prior reports indicate that Re values often remain below this threshold[7].

Solver Settings and Simulation Process: The simulations were performed using a pressure-based solver with the SIMPLE (Semi-Implicit Method for Pressure Linked Equations) algorithm for pressure-velocity coupling. Second-order upwind schemes were used for spatial discretization of momentum and pressure. Convergence was deemed achieved when residuals for continuity and momentum equations fell below 10^{-6} and the mass flow rate at outlets stabilized within 0.1% of the inlet value.

Inlet flow rate determination for the microfluidic scale Carotid Artery-Chip. The *in vivo* scale geometry was initially simulated using the average human common carotid artery (CCA) flow rate to determine its shear rate distribution. The inlet flow rate for the microfluidic chips was then calculated based on its CCA branch's diameter, aiming to achieve a comparable bulk shear rate to the in vivo environment as published (~415 s-1) [3, 4]. Subsequently, the shear rate distribution within the microfluidic scale geometry was inspected. The inlet flow rate was iteratively adjusted to ensure that the overall shear rate distribution closely matched the *in-vivo* condition. This optimized inlet flow rate was then utilized for the experiments.

Post-processing and Analysis. Key hemodynamic parameters were extracted and analyzed, including:

1. Shear rate distribution: Detailed maps of shear rate across the fluid domain were produced.
2. Velocity streamlines: These were generated to visualize flow patterns, particularly in regions of complex geometry.

These CFD results were crucial for validating the flow conditions in our microfluidic devices, ensuring that the scaled-down models accurately replicated the hemodynamic environment of the patient-specific carotid arteries. Regions of interest for the laser injury model were identified based on areas of disturbed flow or elevated shear rate gradients. The shear rate distributions obtained from these simulations were essential for interpreting the results of our thrombosis experiments and drug efficacy studies, allowing us to correlate local hemodynamics with observed thrombotic events.

Alignment and assembly of the PDMS chips

The alignment of the top and bottom halves of the vessel was performed on an Olympus IX83 microscope using the 4X UPlanXApo objective, 0.16NA/13WD. The bottom half chip was first placed on the microscope stage. Then the top half chip was carefully placed on the bottom half chip while visualizing it under the microscope. Further adjustment is optional to precisely align the top and bottom halves.

Supplementary Figures

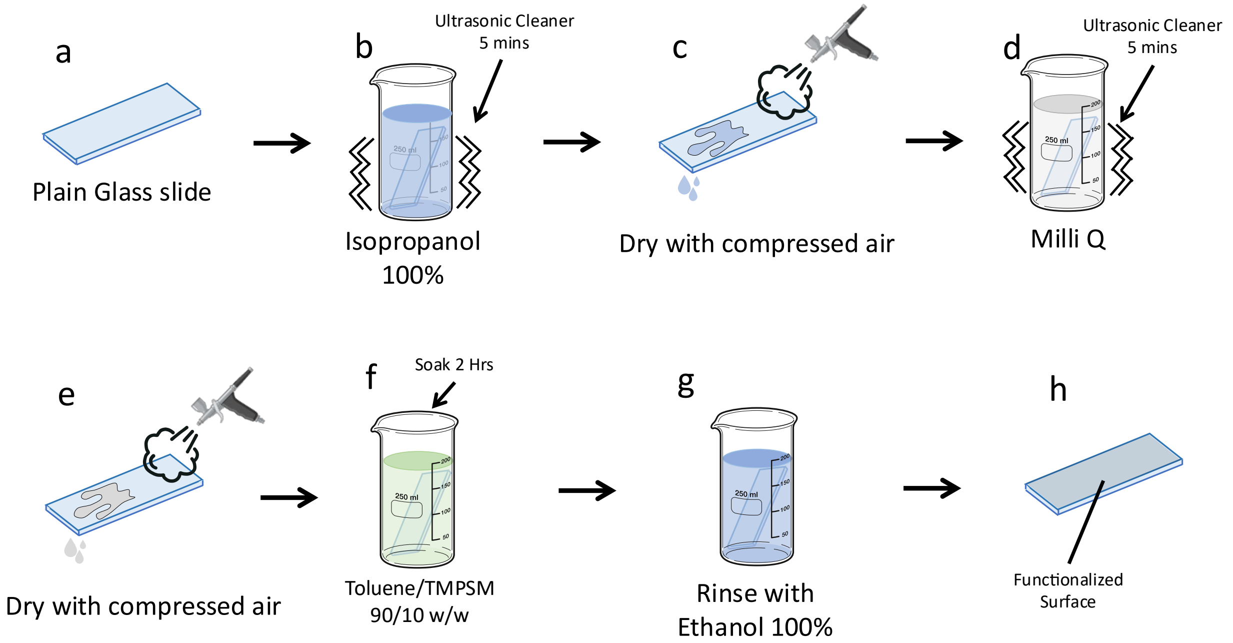

Supplementary Figure S1. Preprocessing of the glass slide to enhance its adhesion to the 3D printing materials. a) A standard plain microscope glass slide without any marks or frost. **b)** The glass slide is first submerged in 100% isopropanol and subjected to 5 minutes of ultrasonic cleaning. **c)** The glass slide is then removed from the isopropanol bath and blown dry with compressed air. **d)** The glass slide is washed in Milli-Q water using an ultrasonic cleaner for 5 minutes. **e)** The glass slide is removed from the Milli-Q bath and blown dry with compressed air. **f)** The glass slide from the previous step is submerged in a solution of Toluene anhydrous (99.8%) and 3-(Trimethoxysilyl) propyl methacrylate (TMSPM, 98%) in a 90/10 w/w ratio for 2 hours. **g)** The glass slide is removed from the solution bath and rinsed with 100% ethanol to remove residual solution. **h)** The surface of the glass slide is now functionalized and ready to be used as a printing substrate.

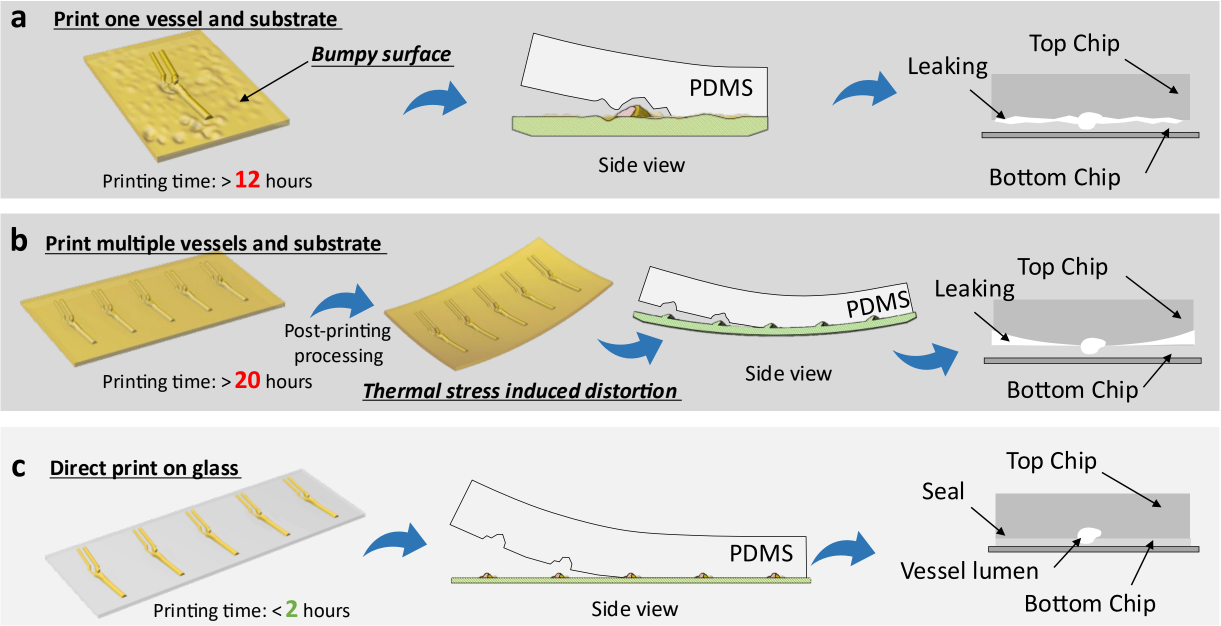

Supplementary Figure S2. Comparison of different printing strategies. a) Print only one vessel mold and its substrate together. The overall printing time will exceed 12 hours, and the imperfections in the top surface will be transferred to the PDMS chip during the casting, which will lead to leaking in the final chip assembly. **b)** Print five vessel molds and their substrates together. Despite of the surface quality issue stated in a) and longer printing time due to larger printing area, the baking in post processing will generate thermal-induced deformation of the substrate, which will cause leakage of the final PDMS chip assembly. **c)** Print five vessel molds on a glass slide substrate. Due to the small printing volume and area, the overall printing time can be reduced to less than 2 hours. The surface finish of the printing will not affect the final PDMS chip assembly.

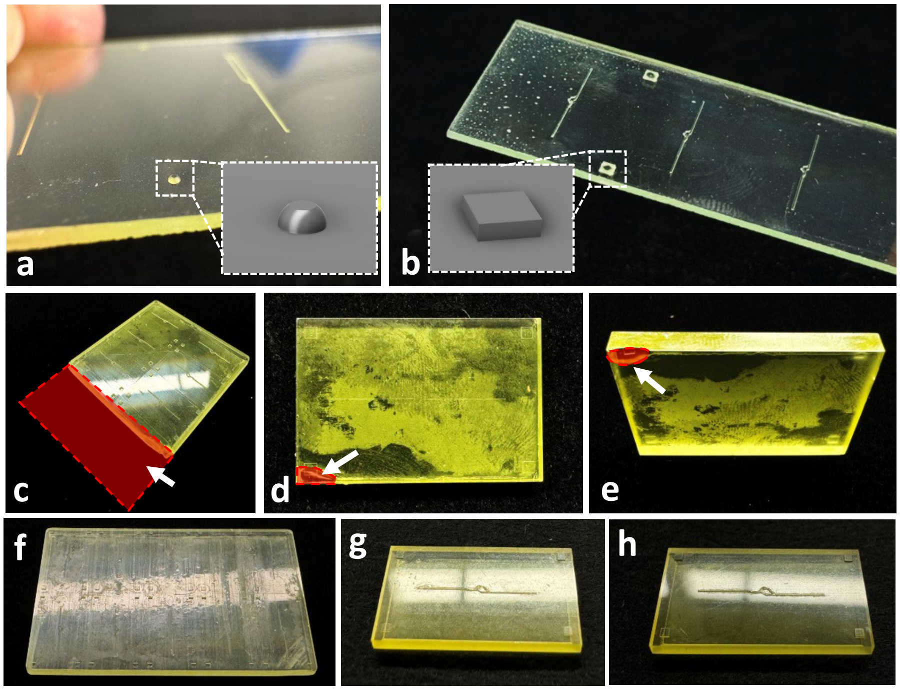

Supplementary Figure S3. Defects and improvements of the printing techniques. a-b) The spacer of the bottom vessel mold is changed from a rectangular **(b)** to a hemispherical **(a)** shape to avoid detachment from the substrate due to PDMS casting. The white colored zone in rectangular block **(b)** represents the detachment from the substrate. A spherical spacer can effectively reduce the detachment from the substrate due to casting. **c-e)** The crack damage when removing printed molds from the build platform using the printing method stated in Figure S2a and S2b, where the red dashed area indicates the damaged region. While there is minor cracked damage near the corner of the mold **(d)** and **(e)**, the whole mold **(c)** cracked into two pieces during the removal of the mold from the 3D printing build platform. **f-h)** The bumpy surface of the printed mold using the printing method stated in Figures S2a and S2b.

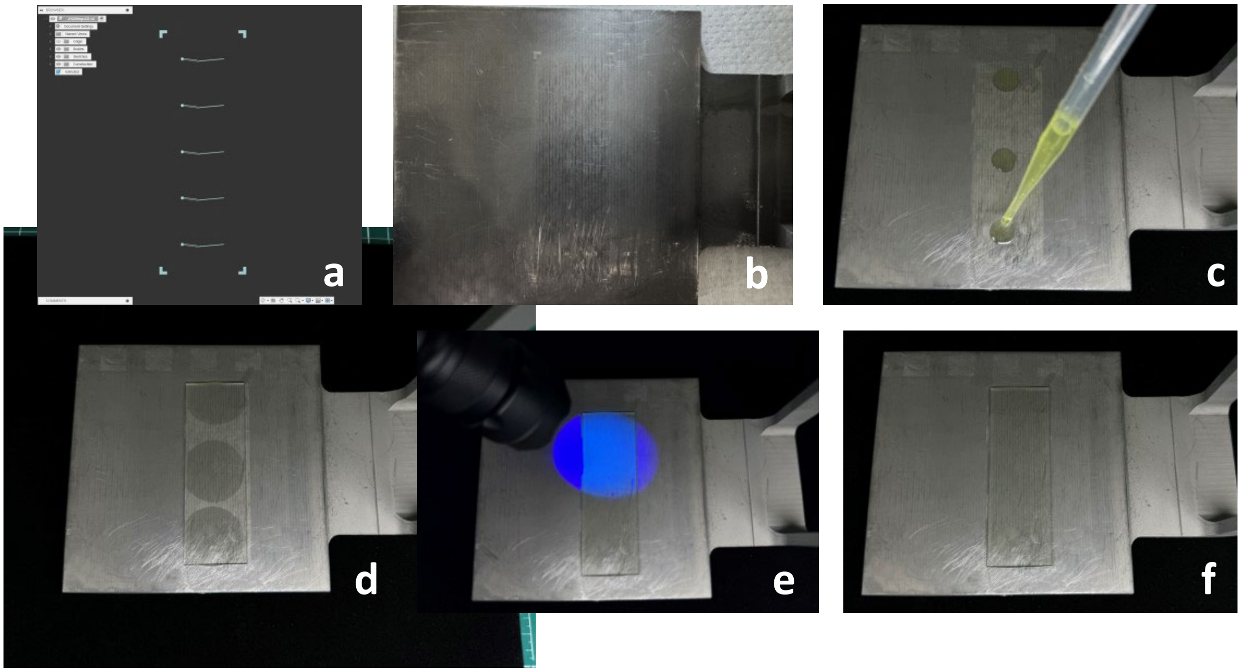

Supplementary Figure S4. Process for precise location and installation of the glass substrate on the 3D print build platform. a) The glass slide locator, composed of four L-shaped brackets, was first designed using CAD software. **b)** Photo shows the L-shaped glass slide locator after being printed on the build platform. **c)** Three drops of printer resin are evenly distributed within the area inside the locator. **d)** The glass slide is precisely placed within the area enclosed by the locators. **e)** The resin is manually cured using a UV torch. **f)** Once fully cured, the glass slide substrate is securely and precisely installed on the build platform and ready for printing.

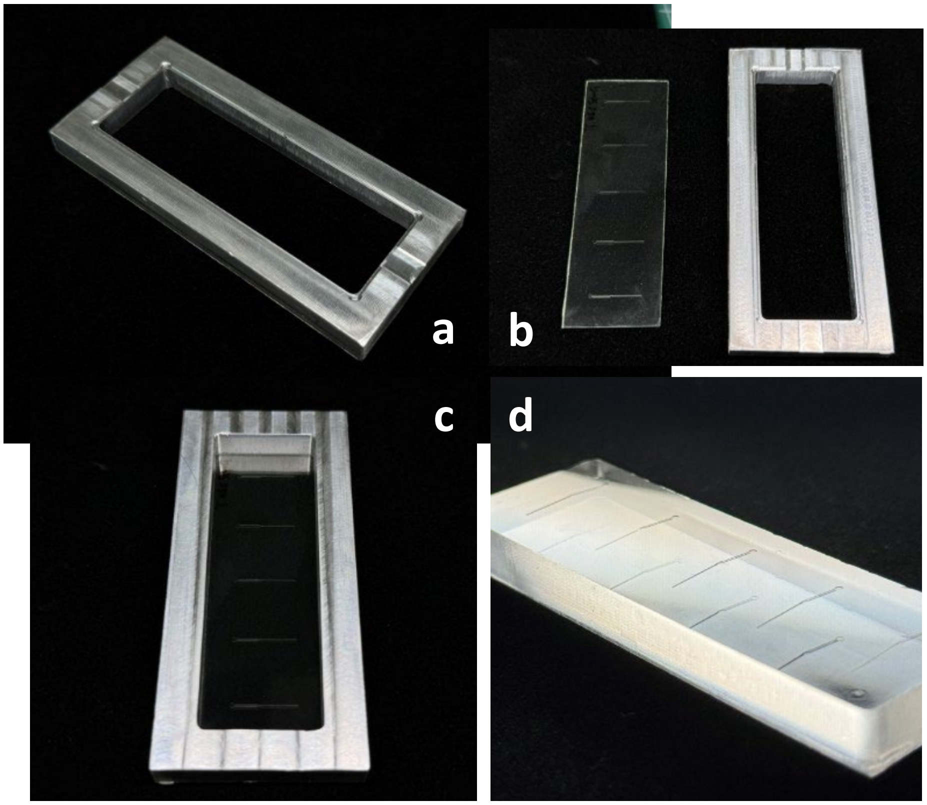

Supplementary Figure S5. The process of making the top half of the PDMS chips. a) The photo of the bottom of the aluminum casting frame. The groves are designed to accurately and securely place the glass slide mold. **b)** The photo showing the glass slide mold with top half vessel geometry printed together with the aluminum casting frame. **c)** The photo shows that the casting frame is installed on the glass slide mold and ready for PDMS casting. **d)** The photo showing the casted PDMS chip contains the top half of the vessel.

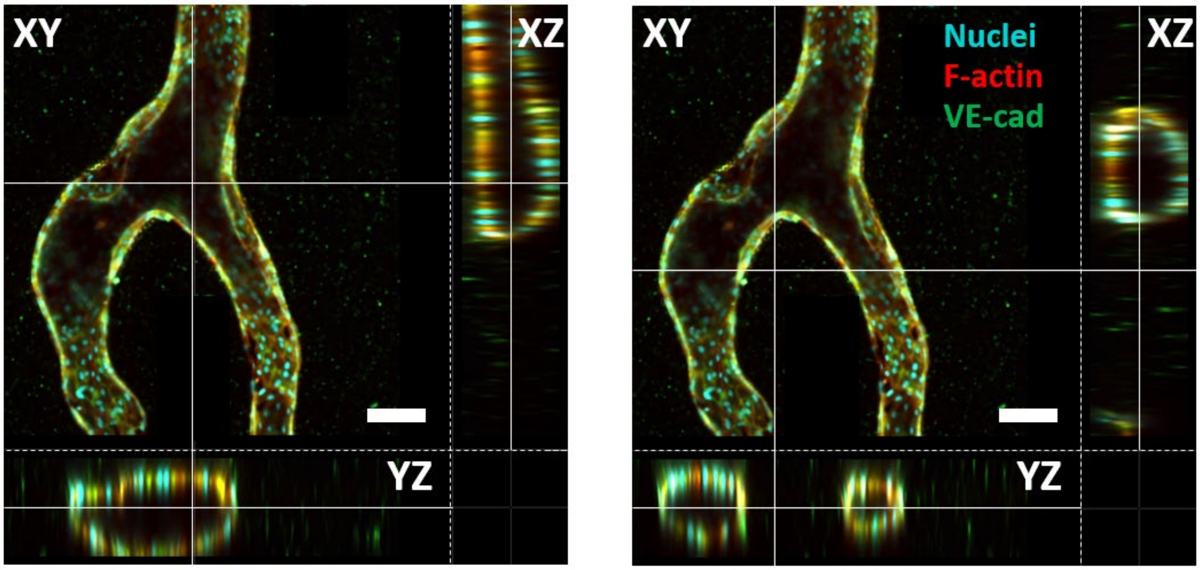

Supplementary Figure S6. Endothelial confluency in the patient-specific Carotid Artery-Chip for Patient #2-G. Endothelial cells were seeded and stained for nuclei (*blue*), F-actin (*red*), and VE-cadherin (*green*), demonstrating confluent monolayer formation and tight intercellular junctions across the vessel lumen. Three orthogonal projections (XY, XZ, YZ) confirm continuous endothelial coverage throughout the vessel circumference, despite optical attenuation caused by the curved microchannel geometry. *Left*: Confocal projections at the carotid sinus (common carotid artery bifurcation zone). *Right*: Projections at the distal bifurcated branches (entering the internal and external carotid arteries) demonstrate similar confluent endothelial layers across both daughter branches. Scale bars: 200 µm.

Supplementary Videos

Video 1
The processes of making a master mold using DLP 3D Printing

Video 2
The process of making the top half of the PDMS chips

Video 3
Precision-punch of inlets and outlets, and alignment of the PDMS chips

Video 4
Assembly, clamping and sterilization of the PDMS chips

Video 5
Comparison of immunostained endothelial layer on the carotid-chip for Patient #1-H with and without TNF-α stimulation through confocal 3D imaging.

Video 6
Comparison of platelet and fibrin accumulation in carotid-chip under whole blood perfusion for Patient #1-H under normal (2mM recalcification) and hypercoagulable (10mM recalcification) blood conditions.

Video 7
Platelet accumulation and translocation in functionalized Carotid-Artery Chip under flow with laser ablation for patient #3-B.

Video 8
Microfluidic Handling and Endothelial Cell Seeding in the Patient-Specific Carotid Artery-Chip.

References

1. Charles Zhao, Y., et al., *Computational Fluid Dynamics Simulations at Micro-Scale Stenosis for Microfluidic Thrombosis Model Characterization.* Molecular & Cellular Biomechanics, 2021. **18**(1): p. 1-10.
2. Zhao, Y.C., et al., *Hemodynamic analysis for stenosis microfluidic model of thrombosis with refined computational fluid dynamics simulation.* Sci Rep, 2021. **11**(1): p. 6875.
3. Forsberg, F., et al., *Shear rate estimation using a clinical ultrasound scanner.* J Ultrasound Med, 2000. **19**(5): p. 323-7.
4. Genkel, V.V., et al., *Association between Carotid Wall Shear Rate and Arterial Stiffness in Patients with Hypertension and Atherosclerosis of Peripheral Arteries.* Int J Vasc Med, 2018. **2018**: p. 6486234.
5. Kerber, C.W. and D. Liepsch, *Flow dynamics for radiologists. II. Practical considerations in the live human.* AJNR Am J Neuroradiol, 1994. **15**(6): p. 1076-86.
6. Lee, S.E., et al., *Direct numerical simulation of transitional flow in a stenosed carotid bifurcation.* J Biomech, 2008. **41**(11): p. 2551-61.
7. Yang, J., et al., *Hemodynamic effects of stenosis with varying severity in different segments of the carotid artery using computational fluid dynamics.* Sci Rep, 2025. **15**(1): p. 4896.
